# Modeling the effects of molecular crowding on cerebellar long term depression

**DOI:** 10.1186/1471-2202-13-S1-P47

**Published:** 2012-07-16

**Authors:** Horace Deans, Fidel Santamaria

**Affiliations:** 1 Department of Biology, The University of Texas at San Antonio, San Antonio, TX 78249, USA

## 

We built a Monte Carlo simulation to study the effects of synaptic content on the expression of long term depression (LTD), a form of synaptic plasticity, in the parallel fiber to Purkinje cell synapse in the cerebellum. LTD is quantified by the reduction in the number of a type of glutamate receptor (AMPAR) in the synapse. Using MCell [1], we modeled the intracellular volume and plasma membrane of a single Purkinje cell dendritic spine. The model tracked the position and biochemical state of all molecules involved in cerebellar LTD after the release of calcium ions (Ca^2+^). Since our model was based on a previously developed well-mixed mass-action model [2], we had to obtain or derive the diffusion coefficients of all molecules under their different biochemical states. When we implemented the Monte Carlo model with the known biophysical properties of molecules, such as translocation and binding, the expression of LTD showed a smooth sigmoidal dependence on the number of released Ca^2+^ which was not observed experimentally. We then implemented the presence of large concentrations of non-reactive macromolecules, a condition known as molecular crowding [3,4]. The non-reactive molecules were modeled using spheres of different sizes either placed regularly or randomly throughout the intracellular space of the spine. A classical approach would suggest that the presence of these macromolecules would result in an increase in the amount of Ca^2+^ needed to induce LTD, producing a rightward shift of the curve. Instead, our simulations show that the steepness of the sigmoidal curve increased, replicating experimental results.

We further studied the effect of molecular crowding on the efficiency of LTD expression. In the control simulation we varied the diffusion coefficient of PKC until no synaptic plasticity was induced. PKC is a key protein in the expression of LTD that phosphorylates AMPARs. We then ran the simulations with increasing presence of crowding molecules. Interestingly, it was at a crowding value of 45% that LTD was not only recovered, but was 30% stronger. Molecular crowding in other cell types has been calculated to be in the order of 40-45 % [5]. Furthermore, theoretical work suggests that molecular crowding could be a mechanism to increase the efficiency of biochemical reactions [6].

In summary, our results show that there is a strong influence of molecular crowding in the activation of biochemical signals in synapses. Based on our simulations we propose that the regulation of crowding molecules in spines could be a mechanism to control the reliability and strength of synaptic plasticity.
